# Mitochondrial genome of *Macrostemum floridum* (Trichoptera)

**DOI:** 10.1080/23802359.2021.1907805

**Published:** 2021-03-31

**Authors:** Hong-lin Qin, Xi-fa Zhong, Yi-min Li, Jing-cai Huang, Hong Wang, Yu-jun Wang

**Affiliations:** a College of Oceanography, Beibu Gulf University, Guangxi, PR China; b College of Life Science and Technology, Guangxi University, Nanning, PR China; c Ocean College, Guangxi Key Laboratory of Beibu Gulf Marine Biodiversity Conservation, Beibu Gulf University, Qinzhou, PR China

**Keywords:** Trichoptera, caddisfly, mitochondrial genome, phylogenetic analysis

## Abstract

Trichoptera are a group of the benthic organism, almost all of which live in water during their life cycle. Trichoptera usually develop through egg, larva, pupa, and moth stages. In its larval stage, Trichoptera usually live in water and are often called the caddisfly. In this study, the mitochondrial genome of *Macrostemum floridum* was analyzed. The total length of the mitochondrial genome is 15,424 bp and consists of 13 protein-coding genes, 22 tRNA genes, 2 rRNA genes, and one control region. The genome has a typical mitochondrial gene sequence of Trichoptera. Phylogenetic analysis of the mitochondrial genomes of 23 species of Trichoptera and Lepidoptera showed that *M. floridum* forms a monophyletic group with other species of Lepidoptera.

*Macrostemum floridum* larva is a medium-sized caddisfly. The whole body is green and the head and chest are bony and oval in shape. The thorax is well developed and there are no gastropods in its abdomen, but there are many brushes, which are used to sense the direction and velocity of the external water flow. *M.acrostemum floridum* larvae spin silk and form funnel-shaped nets in water to filter food in water, including algae, fungi, and organic debris (Wallace et al. 1977; Long et al. 2000). They prefer to live in clean rivers without pollution and *M. floridum* also have certain requirements for water velocity and depth. Therefore, *M. floridum* can be used as an indicator organism to detect and evaluate the water quality (Wallace and Webster 1996; Li and Zhou 2001; Long and Zhang 2002; Huang and Cai 2006; Chen 2013; Lin et al. 2017).

Mitochondria are important markers widely used in the study of genetic diversity, species origin and evolution, and molecular taxonomy. By comparing groups associated with the system development between the change of the length of the mitochondrial genome, tRNA anticodon, or the change of secondary structure, start and stop codon, base composition, preferences, using type, and frequency of codon and rearrangement of the mitochondrial genome characteristics can be for the evolution of different species were analyzed, and determine the evolution of gene sequence (Pan and Bu 2005).

In this study, the complete mitochondrial genome *M. floridum* was sequenced and analyzed, which is helpful to identify the species and locate the evolutionary relationship of *M. floridum*, so that it can be protected and used from a biological perspective.

*M.acrostemum floridum* samples used in this study were collected from Shiwanda Mountain in Guangxi Province, China (108.26E, 22.11N). A specimen has been deposited in the Ocean College Marine Specimen Showroom of Beibu Gulf University of China (voucher No. BBGC 00013). The mitochondrial genome of *M. floridum* was sequenced and assembled using Illumina high-throughput sequencing technology and SPAdes 3.5.0 version software (http://cab.spbu.ru/software/spades/). MITOS (http://mitos.bioinf.uni-leipzig.de/index.py) and ORF (https://www.ncbi.nlm.nih.gov/orffinder/) Finder were used to annotate the mitochondrial genome. The preliminary results were compared with the protein-coding and ribosomal RNA of the mitochondrial genome of related species using BLASTp (https://blast.ncbi.nlm.nih.gov/Blast.cgi) and BLASTn methods.

The total length of the mitochondrial genome is 15,424 bp, including 13 protein-coding genes, 22 tRNA genes, 2 rRNA genes, and one control region. The base composition of the H chain was 39.35% A, 39.52% T, 6.35% G, and 14.78% C, which is similar to that of other Trichoptera insects, and the AT content was higher than that of GC. All tRNA genes except tRNA-Ser (TCT) could be folded into a typical cloverleaf structure with a length of 60–73 bp. 16 s RNA and 12 s RNA genes were located between tRNA-Val and tRNA-Leu and between tRNA-Val and the D-loop, respectively. The initiation codons of 13 protein-coding genes were ATN. The stop codon of the protein-coding genes was an incomplete termination codon T in cox1, cox2, and nad6, and ATN in the remaining genes.

We downloaded 20 genomes of related species from NCBI, including complete or nearly complete mitochondrial genomes (nearly complete refers to complete coding genes) and newly sequenced genomes using RAxML version 8.1.5 software (https://sco.h-its.org/exelixis/web/software/raxml/index.html). The maximum likelihood (ML) method was used to construct a phylogenetic tree and the bootstrap value was set to 1000. *Macrostemum floridum*, along with Hydropsyche, Cheumatopsyche, Potamyia, and Hydromanicus are all a genus of the Hydropsychidae family. According to the phylogenetic tree of *M. floridum*, it can be confirmed that *M. floridum,* Hydropsyche, Cheumatopsyche, etc., are all on the same large branch, but *M. floridum* is different from them, forming a single branch. There is a monophyletic group in the family that includes *M. floridum*, and Phylogenetic analysis showed that *M. floridum* formed sister groups with Hydropsyche and Cheumatopsyche (Figure 1).

**Figure 1.**
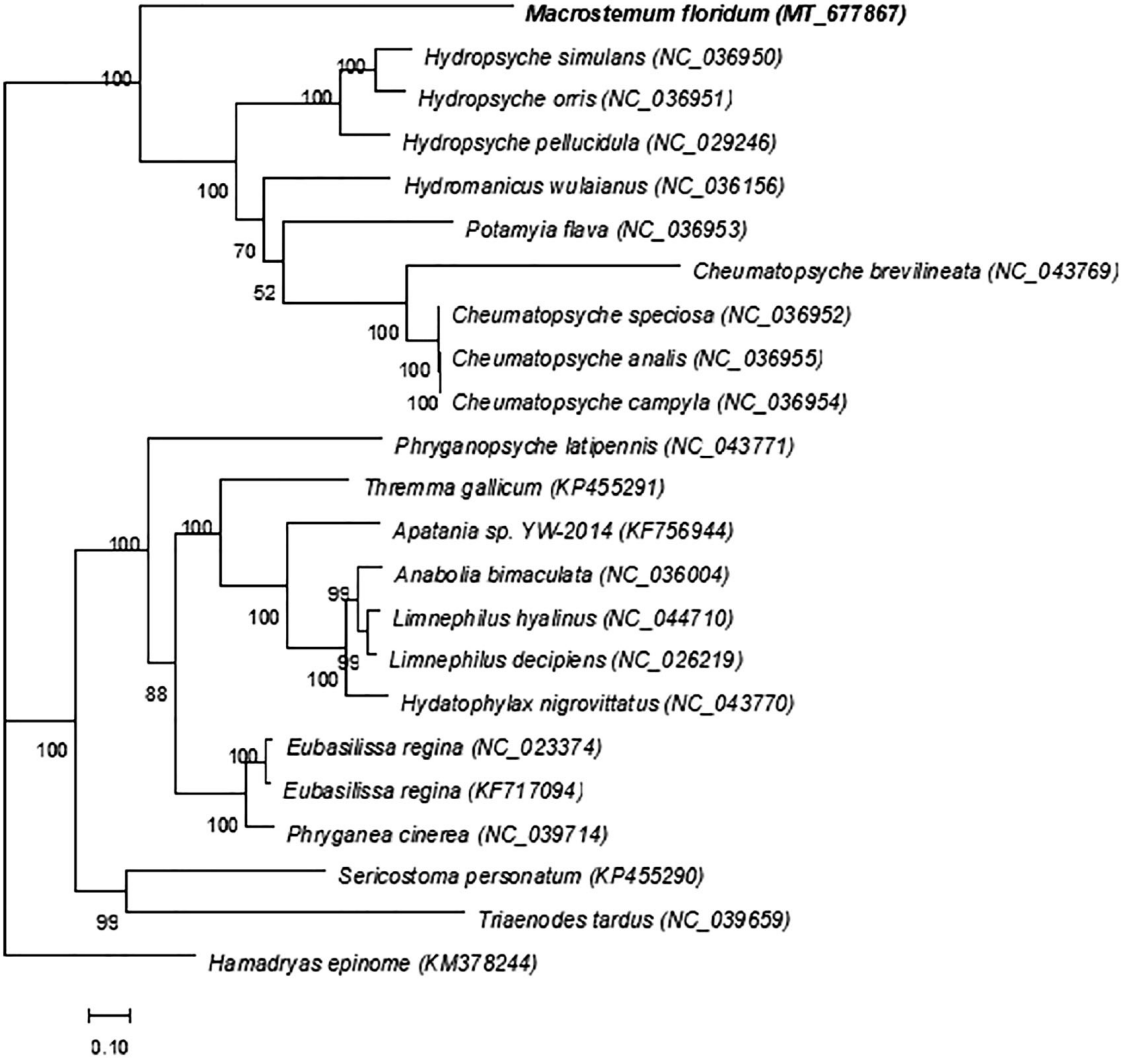
Phylogenetic tree constructed using the maximum likelihood (ML) method.

## Data Availability

The data that support the findings of this study are openly available in GenBank of NCBI at https://www.ncbi.nlm.nih.gov, reference number MT677867.
